# N-acetylcysteine in the Treatment of Internet Gaming Disorder

**DOI:** 10.7759/cureus.28662

**Published:** 2022-09-01

**Authors:** Aakanksha Singh, Gurraj Singh, Satwant Singh, Sana Elham Kazi, Manpreet Gill

**Affiliations:** 1 Psychiatry, Indlas Vijayawada Institute of Mental Health & Neuro Sciences Hospital, Mumbai, IND; 2 Psychiatry, Sri Guru Ram Das Institute of Medical Sciences and Research, Amritsar, IND; 3 Psychiatry, American University of Integrative Sciences, St. Michael, BRB; 4 Psychiatry and Behavioral Sciences, Interfaith Medical Center, Brooklyn, USA

**Keywords:** nac, n-acetylcysteine, gaming addiction, nucleus accumbens, glutamate, dopamine, internet gaming disorder

## Abstract

Internet gaming disorder (IGD) is the persistent and recurrent use of the internet to engage in video gaming through a single or multiplayer interface that can lead to significant impairment or distress. With technological advancements in the last decade via portable handheld devices, along with their global availability, video games have found a new medium in which they can provide instantaneous access for casual and enthusiastic users alike. Unfortunately, this exponentially increases the possibility of addiction. IGD shares a similar pathophysiological etiology to addiction as drugs or gambling. However, it can be challenging to manage IGD due to the ease of video game access and limited understanding of the newly recognized disorder.

This study aims to fill in the knowledge gap concerning the limited research on internet gaming addiction, its consequential effects on human cognitive-behavioral functioning, and pharmacotherapy management as observed in our patient, who developed IGD, starting initially as a casual recreational hobby among peers. This case also highlights the lack of social awareness and seriousness attributed to this disorder. It focuses on using N-acetylcysteine in the management as well as other psychological and psychotropic drugs.

## Introduction

In the modern world, video games have become a routine part of daily activities as a form of leisure and entertainment. In recent years, many technological innovations have been made in the internet and computer gaming field. These changes have transformed the concept of gaming, making it highly addictive for some. As a result, internet gaming disorder (IGD) has recently been included as a potential diagnosis in the fifth edition of the Diagnostic and Statistical Manual of Mental Disorders (DSM-5) by the American Psychiatric Association (APA) [1]. In addition, the World Health Organization (WHO) recognized the impact of gaming on public health. Therefore, a “gaming disorder” diagnosis is mentioned in the 11th revision of the International Classification of Diseases (ICD-11) [2].

The APA defines IGD as persistent and recurrent internet use to engage in games resulting in significant impairment. The WHO defines this disorder as impaired control over gaming, increasing priority given to gaming, and continuing gaming despite negative consequences. The pattern of gaming behavior results in marked distress or significant impairment in several areas of functioning, including, but not limited to, personal, family, social, educational, and occupational. This disorder includes online and offline gaming, digital or video. Online games, such as massively multiplayer online role-playing games (MMORPG), wherein internet users interact with one another, are highly addictive [3].

Neurobiological findings indicate that pathological gambling and drug addiction share a common pathophysiology. There has been a shift in gambling from traditional casinos to online gambling. Online gambling is being promoted and advertised via social media and email marketing. Furthermore, the frequent use of online devices for gambling has been noted in the stock market and bitcoin trading [4]. Online games such as MMORPG are the most addictive. IGD may also correlate with such findings related to addiction. The prevalence of IGD is estimated between 1.2% and 5.5%.

Additionally, one out of ten adolescents playing video games can have problematic gaming use [5]. Hence, there is a need for more therapies to prevent or treat the pathologies related to such addiction. N-acetylcysteine (NAC) is a glutaminergic medication that has been explored as a treatment option to reduce the clinical symptoms among individuals with dependence or addiction [6]. If the pathophysiology of gaming addiction is potentially similar, then similar therapy and intervention may also be beneficial.

We present the case of a college student exhibiting symptoms of IGD and associated addiction behavior. In our case, we review the use of NAC protocol as an intervention to treat IGD, as glutaminergic medications have shown potential in treating substance use disorder and pathological gambling.

## Case presentation

A 19-year-old college student was brought to the outpatient department by his parents for complaints of excessive time spent playing video games. According to the parents, he was engaged in game-play for 12-14 hours daily for the last three months during the coronavirus disease 2019 pandemic while staying in isolation in his bedroom. He reported sleep cycle disruption, irregular meal timing, and neglecting self-care and personal hygiene. He was irritable and verbally aggressive when confronted about his gaming obsession. He stopped showing interest in his routine extracurricular and sports activities and received repeated complaints from his professors about his recent poor academic performance.

He was introduced to online video gaming by his peers two years ago. It initially started with playing for one to two hours during the weekdays and five to six hours during the weekend. As the gaming urge began increasing with tremendous success in the games (i.e., rewards, unlocking achievements, winning streaks, etc.), his playing time rampantly escalated to six to eight hours daily. No symptoms of depression, anxiety, and psychosis were observed with no known family or personal history of psychiatric illness or any substance use disorder.

On Mental Status Examination (MSE), the patient appeared sad and withdrawn with minimal verbal interaction. No formal thought disorder, delusions, obsessions, or suicidal ideas were present. There were no perceptual abnormalities and no cognitive impairment. However, his addictive behavior to video gaming resulted in the impairment of his insight and judgment. IGD was assessed using the nine-item IGD scale. He was then diagnosed with IGD based on the MSE, DSM-5, and IGD scale.

A complete blood count, thyroid, kidney, and liver function test, as well as a urine drug screen, revealed no abnormality.

The patient was moved to the rehabilitation center for three months to provide a gadget-free environment and supportive psychotherapy. Pharmacological treatment consisted of NAC 600 mg twice daily for controlling game-seeking cravings, olanzapine 5 mg once daily to manage aggressive behavior (eventually tapered off within one month and stopped), and diazepam 5 mg at bedtime for insomnia.

During the initial weeks of rehabilitation, the patient demonstrated no significant improvement, with limited insight, was reluctant to share his thoughts, was depressed, and displayed intermittent crying spells. Withdrawal symptoms of intense cravings, restlessness, frequent headaches, and flashbacks of game-playing scenarios in his sleep were frequently displayed. After one month, the patient began exhibiting an understanding of his pathological behavior. His mood and sleep improved, and he reported a reduction in cravings. He started actively participating in indoor/outdoor activities, became more communicative during therapy, and resumed his academic classes.

During a one-month follow-up, the patient and the parents denied witnessing any addiction-seeking behavior or unnecessary indulgence in gaming. He uses his mobile smartphone for routine internet and social media use under parental supervision. He is continuing his medications regularly.

## Discussion

When attempting to explore how video games have the potential for severe addiction, it is essential to understand how repeated neuron stimulation from game-play can affect cortical functioning and cognition. Gray-matter volume reduction has been noted in the anterior cingulate gyrus, dorsolateral prefrontal cortex, and motor cortex in patients with IGD [7].

Alterations in the structure of putamen have been reported in patients with IGD. Functional magnetic resonance imaging analysis has also demonstrated increased activation in the bilateral cingulate cortex, anterior insula, and striatum [8]. Impaired cognitive control in patients with IGD leading to compulsive gaming behavior may be related to increased striatum volume. The amygdala is responsible for emotional control, and impairment in its function may be associated with an enhanced emphasis on emotions and immediate rewards, leading to extreme gaming.

The mesolimbic dopamine pathway is the intrinsic reward circuit pathway. It includes dopaminergic projections from the ventral tegmental area to the nucleus accumbens and olfactory tubercle. Similar to substance use disorders, a reduction in dopaminergic activity associated with a deficiency in overall reward results in excessive internet use and gaming [9].

Glutamate is the primary excitatory neurotransmitter of the nervous system. Addiction results from a reduced ability to impede drug-seeking desire in response to environmental constraints. The proposed mechanism is an alteration in the glutamate homeostasis and activation of the N-methyl-D-aspartate and dopamine glutamatergic receptors. In patients with substance use, blocking the release of glutamate prevents drug-seeking behaviors [10].

Glutamatergic medications act concurrently on dopaminergic and glutamatergic systems and help prevent relapse in patients with substance use. NAC, a glutamatergic drug, comes from the amino acid L-cysteine and can increase the extracellular levels of glutamate in the nucleus accumbens. It is effectively used in treating substance use disorder and trichotillomania and may be beneficial in treating obsessive-compulsive disorder and gambling disorder. Glutamate acts at the metabotropic and the ionotropic glutamate receptors. NAC may stimulate inhibitory metabotropic glutamate receptors, reducing the synaptic release of glutamate and helping manage drug and behavioral addictions [11].

There are no long-term follow-up studies for the treatment of IGD. However, early diagnosis of IGD can prevent detrimental effects on functional and social areas of life. Management should be undertaken with utmost diligence due to the potential implications of withdrawal features, anxiety, depression, and negative self-esteem, along with addiction-seeking behavior through other forms of “reward.”

## Conclusions

This case report highlights the lack of social awareness, understanding, and consequential impact of IGD on an individual’s life while proposing using NAC as a novel treatment approach for IGD. Though there is limited availability of data on the use of NAC in treating addictions, including gambling disorders, further research can shed light on its potentially widespread use while bringing forth the implications of gaming addiction and its rampant effects on social and mental health.
